# Minimally invasive surgery reduces the risk of loss of independence after pancreatoduodenectomy in elderly patients

**DOI:** 10.1007/s00464-025-12518-2

**Published:** 2025-12-29

**Authors:** Atsushi Nara, Hiroki Ueda, Yukue Shimizu, Daisuke Asano, Yoshiya Ishikawa, Keiichi Akahoshi, Eriko Katsuta, Junko Torigoe, Daisuke Ban

**Affiliations:** 1 Department of Hepatobiliary and Pancreatic Surgery, Graduate School of Medicine, Institute of Science Tokyo, 1-5-45, Yushima, Bunkyo-Ku, Tokyo, 113-8519 Japan; 2 Department of Clinical Nutrition, Institute of Science Tokyo Hospital, 1-5-45 Yushima, Bunkyo-ku Tokyo, 113-8519 Japan

**Keywords:** Pancreatoduodenectomy, Sarcopenia, Minimally invasive surgery, Elderly, Loss of independence

## Abstract

**Objectives:**

Pancreatoduodenectomy (PD) is a highly invasive surgery that raises concerns about postoperative loss of independence (LOI), a critical outcome defined as a decline in activities of daily living (ADL). LOI reflects a significant shift in functional status, often requiring additional care such as rehabilitation or home-based healthcare. While reducing complications and mortality is prioritized, maintaining a preoperative lifestyle remains underexplored. Therefore, this study aimed to elucidate the risk factors of LOI after PD.

**Methods:**

We retrospectively analyzed 215 patients underwent PD between August 2017 and April 2024. Patients were classified into young (< 65 years) and elderly groups (≥ 65 years). Using univariate and multivariate analyses, we assessed risk factors of LOI after PD.

**Results:**

There was no incidence of LOI in the young group, whereas 22 patients (16.7%) developed LOI in the elderly group. Univariate analysis revealed that age ≥ 80 years (*P* < 0.001), sarcopenia (*P* < 0.001), open surgery (*P* = 0.013), and malignant disease (*P* = 0.009) were the risk factors of LOI. Multivariate analysis identified age ≥ 80 years (*P* < 0.001), sarcopenia (*P* < 0.001), and open surgery (*P* = 0.040) as the independent risk factors of LOI in the elderly group. Using these three factors, we established LOI score. This LOI score significantly correlated with the incidence of LOI (*P* < 0.001).

**Conclusions:**

This is the first study to identify the risk factors of LOI after PD. It may help the decision-making regarding MIS surgery with other risk factors in clinical practice.

**Graphical abstract:**

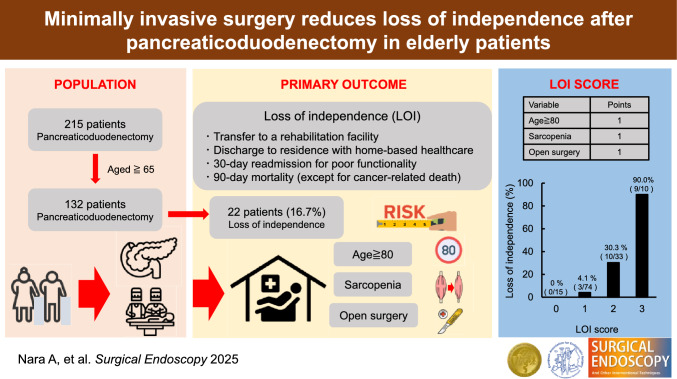

**Supplementary Information:**

The online version contains supplementary material available at 10.1007/s00464-025-12518-2.

In recent years, the number of elderly patients undergoing pancreatoduodenectomy (PD) has increased owing to the aging population [1]. PD is a highly invasive surgery that is mainly performed for pancreatic head and biliary tumors. Postoperative complications such as pancreatic fistula, delayed gastric emptying, and postoperative bleeding are major concerns [2, 3]. On the other hand, elderly patients are inherently frail and may have conditions such as sarcopenia, which makes them more vulnerable to postoperative complications that require additional attention.

With the increase in long-term survival after PD, greater attention is being paid to mid- and long-term outcomes, such as Quality of Life (QOL) and Activities of Daily Living (ADL) [4], rather than short-term complications alone. A distinct concept of decrease in ADL and QOL is loss of independence (LOI), which refers to functional impairment resulting in loss of autonomy and inability to sustain social engagement. In the current clinical landscape, LOI is gaining increasing importance [5–8]. As an outcome-focused and comprehensive measure, LOI reflects the real-world impact of postoperative decline more accurately. Although there have been reports on ADL impairment after gastrointestinal surgeries or hepatic and pancreatic resections [9, 10], no study has focused on LOI following PD. Since surgeries for elderly patients are increasing, evaluating the risk of LOI is particularly valuable to optimize perioperative care and support shared decision-making. By predicting LOI, it is possible to implement appropriate perioperative management and discharge planning, resulting in facilitating smoother transitions to home healthcare or rehabilitation facilities, reducing hospitalization duration, minimizing costs, and preventing readmission due to unanticipated LOI. Therefore, we conducted a retrospective study on PD patients, analyzing the risk factors for postoperative LOI and elucidating its clinical characteristics.

## Materials and methods

### Study design

**Fig. 1:**
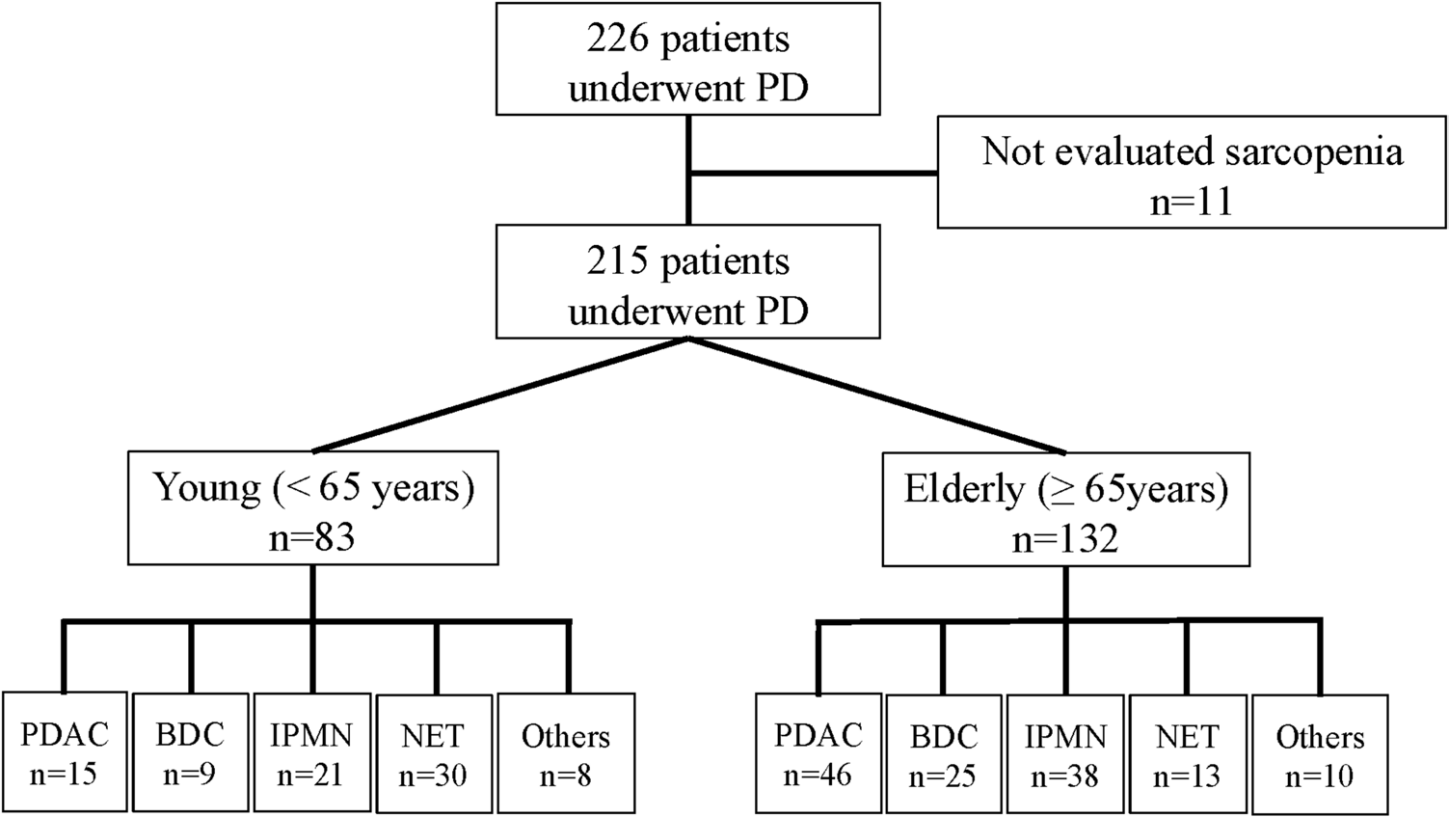
Primary disease in young and elderly group patients *PD* Pancreaticoduodenectomy, *PDAC* Pancreatic ductal adenocarcinoma, *BDC* Bile duct cancer, *IPMN* Intraductal papillary mucinous neoplasm, *NET* Neuroendocrine tumor

This study was a single-center, retrospective study. Of the 226 patients who underwent PD between August 2017 and April 2024, 11 patients who were not evaluated for sarcopenia preoperatively were excluded. The remaining 215 patients were included in the final analysis. Our study period was selected to include all consecutive cases after the introduction of standardized preoperative functional assessments, including nutritional and sarcopenia evaluations using the InBody, at our institution. Statistically, the study was continued through 2024 to ensure the accumulation of a sufficient sample size, with more than 100 PD cases included, allowing for meaningful analysis. According to the World Health Organization (WHO) definition, 132 patients aged ≥ 65 years were analyzed as elderly (Fig. 1). All patients were independent before surgery. Independence was defined as sufficient activities of daily living and no requirement of home medical or long-term care insurance. Clinical information was obtained from the electronic patient record system in the hospital. This study was approved by the Committee on Life Sciences and Bioethics of the Institute of Science Tokyo (#2000–1080-1).

#### Definition

**Fig. 2:**
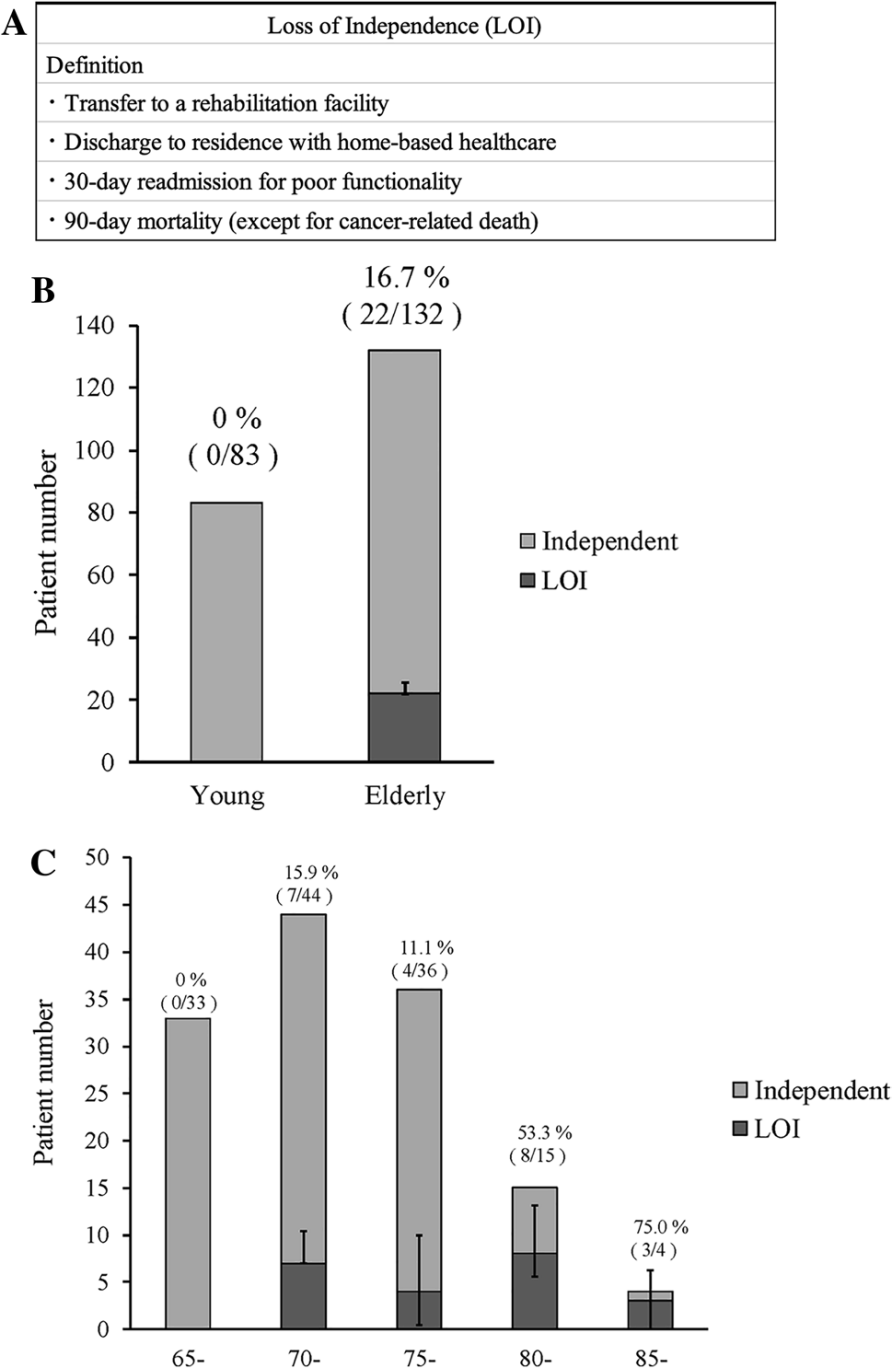
A: LOI definition. B: Incidence of LOI in young and elderly group. C: Incidence of LOI by age

In this study, LOI after PD was defined as either introduction of home-based healthcare, rehabilitation transfer, readmission within 30 days due to poor functional status, or 90-day mortality (except for cancer-related deaths) (Fig. 2A) [11]. Home-based healthcare is defined as the introduction of two services: home nursing and home care visits. Medical care for elderly individuals living at home or in medical facilities is covered by the Long-term Care Insurance System, which is a part of universal insurance. All Japanese individuals aged ≥ 40 years pay insurance premiums, and those aged ≥ 65 years are eligible to receive Long-term Care Insurance System. Length of stay was defined as the days from surgery to discharge. In some cases, LOI resulted in extended hospitalization due to need for additional time to arrange transfers for rehabilitation or preparation for home-based healthcare. When deciding on discharge, unmeasured confounders such as socioeconomic status or baseline caregiver support are also considered by a multidisciplinary team.

InBody S10 (Biospace Inc., Tokyo, Japan) is a device that can conveniently analyze body composition using the Bioelectrical Impedance Analysis (BIA) method to measure muscle quantitatively [12]. The measurement takes approximately 90 s and involves the attachment of electrodes to the limbs. This allows measurement in standing, sitting, or supine positions. The patients who underwent PD were preoperatively assessed muscle strength and grip strength using InBody S10 and a digital grip strength meter. Sarcopenia was defined by skeletal muscle index and grip strength. Patient with below the criteria set by the Asian Sarcopenia Working Group, i.e. skeletal muscle mass index < 7.0 kg/m^2^ for males and < 5.7 kg/m^2^ for females, as well as grip strength < 28 kg for males and < 18 kg for females [13, 14], was diagnosed as sarcopenia.

## Surgical procedure

PD was performed using a subtotal stomach-preserving pancreaticoduodenectomy technique, preserving over 95% of the stomach while removing the pylorus. Reconstruction was performed using the Child method with Braun anastomosis. We are also introducing minimally invasive (laparoscopic or robot-assisted) PD starting in 2018 and the number of cases has been increased over the time (Fig. S1). Minimally invasive PD is applied to both benign and malignant tumors, provided that curative resection is technically achievable and no combined vascular or multivisceral resection is required. Our hospital is accredited as a “High-Volume Center A” by the Japanese Society of Hepato-Biliary-Pancreatic Surgery. This accreditation requires an annual volume of at least 50 pancreaticoduodenectomies or other complex hepato-biliary-pancreatic operations, thereby ensuring institutional expertise. The surgical team consisted of experienced attending surgeons (11 for open PDs and 5 for minimally invasive PDs), and all procedures involved board-certified expert or instructor surgeons of the society, ensuring consistently high surgical quality. Importantly, minimally invasive procedures were conducted by surgeons certified by the Japan Society for Endoscopic Surgery (JSES) through the Endoscopic Surgical Skill Qualification System. This certification requires the completion of at least 20 advanced laparoscopic procedures and the successful passage of a rigorous peer-reviewed video assessment, reflecting a high level of technical expertise and standardization. We combined the laparoscopic and robotic approaches into one group because they share similar operative principles and incision sizes [15, 16].

## Postoperative complications

Postoperative pancreatic fistula (POPF) was defined as clinically relevant POPF based on the International Study Group of Pancreatic Surgery (ISGPS) criteria [17]. Delayed gastric emptying (DGE) was also defined as grade B or C, according to the criteria proposed by ISGPS [18]. Other complications were defined according to the Clavien-Dindo classification.

## Statistical analysis

Categorical variables were summarized as numbers and percentages, and comparison between groups were performed using Fisher's exact test, as appropriate. Continuous variables were summarized as medians, and comparisons between groups were conducted using the Mann–Whitney *U* test. The Cochran-Armitage trend test was applied to categorical variables to stratify the groups based on the number of corresponding risk factors and assess the trend in LOI incidence. Multiple regression analysis was performed for multivariate analysis. No significant multicollinearity was observed based on the variance inflation factor (VIF) values. Receiver operating characteristic (ROC) curves [sensitivity and 1-specificity] were created, and the area under the ROC curve (AUC) and its 95% confidence interval (CI) were calculated. Variables with a *P* < 0.05 in univariate analysis were included in the multivariate analysis. Statistical significance was set at *P* < 0.05. All analyses were conducted using a complete-case approach, as no data were missing in the variables included in the multivariable models. All statistical analyses were performed using EZR (Saitama Medical Center, Jichi Medical University, Saitama, Japan), a graphical user interface for R (The R Foundation for Statistical Computing, Vienna, Austria) [19].

## Results

### Patient demographics

Among 215 patients (121 males and 94 females), 132 patients were aged ≥ 65 years and remaining 83 were younger than 65 years, classified as elderly and young groups, respectively (Table 1, Fig. 1). The prevalence of sarcopenia was higher in the elderly group, as expected (6.0% vs. 32.6%, *P* < 0.001). The elderly had a higher proportion of malignant diseases than the younger group (28.9% vs. 55.3%, P < 0.001). (Table 1, Fig. 1).

**Table 1:** Clinical demographics and surgical outcomes in the patients underwent pancreatoduodenectomy

Variables	Young (< 65 years) (*n* = 83)	Elderly (≥ 65 years) (*n* = 132)	*P*
*Patient demographics*			
Age, years; median (range)	56 (21–64)	73 (65–88)	< 0.001
Sex, male (%)	41(49.4)	80(60.6)	0.121
BMI, kg/m^2^; median (range)	23.3 (15.4–36.7)	21.6 (15.3–29.8)	0.057
ASA score ≥ 3 (%)	6 (7.2)	23 (17.6)	0.040
Sarcopenia (%)	5 (6.0)	43 (32.6)	< 0.001
Primary disease			
Malignancy (%)	24 (28.9)	73 (55.3)	< 0.001
Pancreatic cancer (%)	15 (18.1)	46 (34.8)	
Cholangiocarcinoma (%)	6 (7.2)	14 (10.6)	
Pancreatic neuroendocrine tumor (%)	30 (36.1)	13 (9.8)	
Intraductal papillary mucinous neoplasms (%)	21 (25.3)	38 (28.8)	
Papillary carcinoma (%)	3 (3.6)	11 (8.3)	
Others (%)	8 (9.6)	10 (7.6)	
*Preoperative factors*			
Preoperative blood test			
WBC (/㎕)	5300 (2200–10200)	5250 (2200–9900)	0.921
Lymphocyte (/㎕)	1452 (535–2815)	1372 (414–2867)	0.360
Albumin (g/dL)	4.2 (2.7–4.9)	4.0 (2.2–4.8)	< 0.001
CRP (mg/dl)	0.05 (0.02–8.29)	0.09 (0.02–3.35)	0.051
Prognostic nutritional index (PNI)	49.8 (33.0–56.8)	46.7 (32.3–56.8)	< 0.001
Neoadjuvant chemotherapy (%)	9 (10.8)	17 (12.9)	0.830
*Surgery related factors*			
MIS/Open	21/62	24/108	0.231
Portal vein resection (%)	6 (7.2)	12 (9.1)	0.802
Colon resection (%)	1 (1.2)	4 (3.0)	0.651
Operation time, min; median (range)	493 (281–935)	499 (289–876)	0.995
Blood loss, ml; median (range)	400 (14–3988)	450 (40–5971)	0.209
Blood transfusion (%)	5 (6.0)	10 (7.6)	0.134
*Postoperative complications*			
Clavien-Dindo classification ≥ grade IIIa (%)	30 (36.1)	42 (31.8)	0.554
POPF (%)	29 (34.9)	39 (29.8)	0.454
DGE (%)	10 (12.0)	10 (7.6)	0.336
Post pancreatectomy hemorrhage (%)	5 (6.0)	11 (8.3)	0.603
Hospital stays, days; median (range)	23 (12–116)	24 (13–109)	0.503

*WBC* White Blood Cell counts, CRP: C-reactive protein, BMI: body mass index. ASA: American Society of Anesthesiologist's physical status, PNI: prognostic nutritional index, MIS: minimally invasive surgery, POPF: postoperative pancreatic fistula, DGE: delayed gastric emptying

## LOI incidence and risk factor in the elderly group

No patient developed LOI in the young group, whereas, among 132 elderly group patients, 22 (16.7%) developed LOI (Fig. 2B). LOI occurred in 0% (0/33) of patients aged 65–69, 15.9% (7/44) in those aged 70–74, 11.1% (4/36) in those aged 75–79, 53.3% (8/15) in those aged 80–84, and 75.0% (3/4) in those aged 85 and older (Fig. 2C). The breakdown of LOI reasons included 17 cases requiring home-based healthcare, two cases transferred to rehabilitation facilities, and three cases readmitted due to systemic deterioration within 30 days (Table S1).

We proceeded to investigate the risk factor for postoperative LOI in the elderly group. Analysis using the ROC curve identified a cutoff of 79 years for LOI risk; therefore, age ≥ 80 years was defined as a risk factor (Fig. S2). Univariate analysis revealed that age ≥ 80 years (7.3% vs. 50.0%, *P* < 0.001), sarcopenia (23.6% vs. 77.3%; *P* < 0.001), malignant disease (50.0% vs. 81.8%; *P* = 0.009), and open surgery (78.2% vs. 100.0%; *P* = 0.013) were the risk factors for postoperative LOI (Table 2). Factors such as underweight, neoadjuvant chemotherapy, POPF, and portal vein resection were not significantly associated with LOI. The length of hospital stay was significantly longer in the LOI group than in the non-LOI group (23 vs. 33 days; *P* = 0.018). Multivariate analysis identified age ≥ 80 years (regression coefficient [β]: 0.40; 95% CI: 0.24–0.55; *P* < 0.001), sarcopenia (β: 0.27; 95% CI: 0.15–0.38; *P* < 0.001), and open surgery (β: 0.15; 95% CI: 0.01–0.28; *P* = 0.040) as independent risk factors (Table 2).

**Table 2:** Univariate and multivariate analyses for the risk of loss of independence after pancreatoduodenectomy in the Elderly group

		Univariate Analysis				Multivariate Analysis		
Variables		Independence (*n* = 110)	Loss of Independence (*n* = 22)	P		regression coefficient	(95% CI)	P
*Patient characteristics*								
Age, years (%)	> 80	8 (7.3)	11 (50.0)	< 0.001		0.40	0.24–0.55	< 0.001
Sex (%)	male	65 (59.1)	15 (68.2)	0.482				
BMI, kg/m2	< 18.5	13 (11.8)	6 (27.3)	0.090				
ASA classification (%)	III-IV	18 (16.5)	5 (22.7)	0.540				
Sarcopenia (%)	Yes	26 (23.6)	17 (77.3)	< 0.001		0.27	0.15–0.38	< 0.001
Primary disease								
Malignancy	Yes	55 (50.0)	18 (81.8)	0.009		0.07	0.04–0.06	0.239
Pancreatic cancer		37	9					
Cholangiocarcinoma		9	5					
Pancreatic neuroendocrine tumor		12	1					
Intraductal papillary mucinous neoplasms		36	2					
Papillary carcinoma		9	2					
Others		7	3					
*Preoperative factors*								
Preoperative Blood test								
WBC (/㎕)	> 8500	4(3.6)	1 (4.5)	> 0.999				
Lymphocyte (/㎕)	< 1500	73 (66.4)	11 (50.0)	0.154				
Albumin (g/dL)	< 3.5	18 (16.4)	7 (31.8)	0.132				
CRP (mg/dl)	> 1.0	5 (4.5)	1 (4.5)	> 0.999				
Prognostic nutritional index (PNI)	< 45	43 (39.1)	13 (59.1)	0.101				
Neoadjuvant chemotherapy	Yes	14 (12.7)	3 (13.6)	> 0.999				
*Surgery related factors*								
MIS/Open	Open	86 (78.2)	22 (100.0)	0.013		0.15	0.01–0.28	0.040
Portal vein resection (%)	Yes	9 (8.2)	3 (13.6)	0.421				
Colon resection (%)	Yes	2 (1.8)	2 (9.1)	0.129				
Operation time, min	> 600	26 (23.9)	4 (18.2)	0.782				
Blood loss, ml	> 1500	4 (3.7)	2 (9.1)	0.265				
Blood transfusion (%)	Yes	9 (8.3)	1 (4.5)	> 0.999				
*Postoperative complications*								
Clavien-Dindo classification (%)	** ≥ **grade IIIa	31 (28.2)	11(50.0)	0.077				
POPF (%)	Yes	28 (25.5)	10 (45.5)	0.073				
DGE (%)	Yes	7 (6.4)	3 (13.6)	0.369				
Post pancreatectomy hemorrhage (%)	Yes	9 (8.3)	1 (4.5)	> 0.999				
Hospital stays, days; median (range)		23 (13–109)	33 (16–78)	0.018				

*WBC* White Blood Cell counts, *CRP* C-reactive protein, *CI* confidence interval, *BMI* body mass index, *ASA* American Society of Anesthesiologist's physical status, *PNI* prognostic nutritional index, *MIS* minimally invasive surgery, *POPF* postoperative pancreatic fistula, *DGE* delayed gastric emptying

**Fig. 3:**
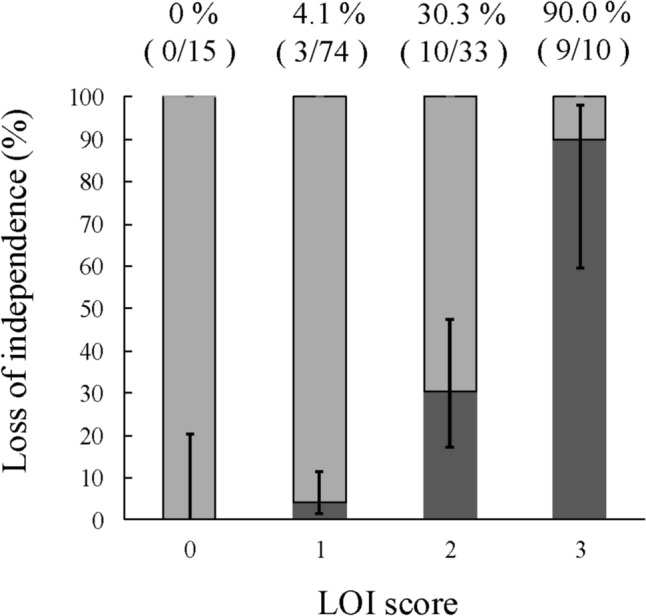
The incidence of loss of independence after PD with LOI score

Therefore, we proposed the LOI score with these three factors: age ≥ 80 years, sarcopenia, and open surgery. Each factor has 1 point, and the total point is calculated by adding all points. The LOI incident rates in the scores 0, 1, 2, and 3 were 0% (0/15 cases), 4.1% (3/74 cases), 30.3% (10/33 cases), and 90.0% (9/10 cases) (Fig. 3). As expected, there was a significant correlation between this score and the LOI incidence rate (Cochran–Armitage trend test, *P* < 0.001). The incidence of postoperative LOI was higher as the number of risk factors was higher. ROC curve analysis using this score for LOI prediction showed an AUC of 0.873 (95% CI: 0.794–0.951) (Fig. S3).

## Patient characteristics of minimally invasive surgery (MIS)

To clarify the potential benefits of minimally invasive surgery (MIS) in elderly patients, we further analyzed the patient characteristics of MIS patients in the elderly group. Among the 132 elderly patients, 24 (18.2%) underwent MIS. There were no significant differences between the MIS and open surgery groups in the proportion of patients age ≥ 80 (15.7% vs. 8.3%; *P* = 0.524), sex (male: 60.2% vs. 62.5%; *P* > 0.999), BMI (22.0 vs. 21.6; *P* = 0.547), the proportion of preoperative American Society of Anesthesiologist's physical status (ASA) scores ≥ 3 (16.8% vs. 20.8%; *P* = 0.767), or sarcopenia (33.3% vs. 29.2%; *P* = 0.812). Despite these similar baseline characteristics, the incidence of LOI was significantly lower in the MIS group compared to the open surgery group (20.4% vs. 0.0%; *P* = 0.013) (Table 3). Similar results were obtained even when the analysis was limited to the period after the introduction of MIS. (Table S2).

**Table 3:** Clinical characteristics and surgical outcomes in the elderly patients comparing Open Surgery (*n* = 108) vs MIS (Laparoscopic *n* = 12, Robotic *n* = 12)

Variables	Open Surgery (*n* = 108)	MIS (*n* = 24)	*P*
*Patient characteristics*			
Age, years; median (range)	74 (65–88)	71.5 (65–82)	0.057
Age ≥ 80 years	17 (15.7)	2 (8.3)	0.524
Sex (male/female)	65/43	15/9	> 0.999
BMI, kg/m^2^; median (range)	22.0 (15.8–29.8)	**23.3** (15.3–28.8)	0.547
ASA score ≥ 3 (%)	18 (16.8)	5 (20.8)	0.767
Sarcopenia (%)	36 (33.3)	7 (29.2)	0.812
Primary disease			
Malignancy (%)	65 (60.2)	8 (33.3)	0.023
Pancreatic cancer	42 (38.9)	4 (16.7)	
Cholangiocarcinoma	13 (12.0)	1 (4.2)	
Pancreatic neuroendocrine tumor	8 (7.4)	5 (20.8)	
Intraductal papillary mucinous neoplasms	29 (26.9)	9 (37.5)	
Papillary carcinoma	8 (7.4)	3 (12.5)	
Others	8 (7.4)	2 (8.3)	
*Preoperative factors*			
Preoperative blood test			
WBC (/㎕)	5500 (2500–9900)	5150 (3200–7100)	0.074
Lymphocyte (/㎕)	1379 (569–2867)	1327 (414–2819)	0.385
Albumin (g/dL)	3.9 (2.2–4.8)	4.1 (3.3–4.6)	0.020
CRP (mg/dl)	0.10(0.02–3.35)	0.04 (0.02–3.35)	0.005
Prognostic nutritional index (PNI)	46.5 (32.3–56.8)	47.1 (40.4–54.1)	0.169
Neoadjuvant chemotherapy (%)	15 (13.9)	2 (8.3)	0.524
*Surgery related factors*			
Portal vein resection (%)	12 (11.1)	0 (0)	0.122
Colon resection (%)	4 (3.7)	0 (0)	> 0.999
Operation time, min; median (range)	477 (289–823)	499 (383–876)	< 0.001
Blood loss, ml; median (range)	525 (115–5971)	238 (40–1276)	< 0.001
Blood transfusion (%)	10 (9.3)	0 (0)	0.385
*Postoperative complications*			
Clavien-Dindo classification ≥ grade IIIa (%)	35 (32.4)	7 (29.2)	0.814
POPF (%)	32 (29.9)	7 (29.2)	> 0.999
DGE (%)	8 (7.4)	2 (8.3)	> 0.999
Post pancreatectomy hemorrhage (%)	8 (7.4)	3 (12.5)	0.420
Hospital stays, days; median (range)	24 (14–109)	26 (13–59)	0.906
Loss of independence (%)	22 (20.4)	0 (0.0)	0.013

*MIS* minimally invasive surgery, *WBC* White Blood Cell counts, *CRP* C-reactive protein, *BMI* body mass index, *ASA* American Society of Anesthesiologist's physical status, *PNI* prognostic nutritional index, *POPF* postoperative pancreatic fistula, *DGE* delayed gastric emptying

## Discussion

We investigated risk factors of LOI after PD in elderly patients. To our knowledge, this study is the first report to assess the risk of LOI after PD. In our cohort, nearly one in six elderly patients developed LOI, which is an unignorable proportion. Among various potential factors, age ≥ 80 years, sarcopenia, and open surgery were identified as significant independent risk factors. Conversely, expected risk factors such as malnutrition, malignancy, neoadjuvant chemotherapy, portal vein resection, POPF, operative time, and bleeding volume were not independently associated with LOI. Notably, the length of postoperative hospital stay was not directly related to LOI incidence, as some patients developed LOI despite early discharge, most likely due to decline in ADL, resulting in readmission. The most common cause of LOI was the introduction of home-based healthcare, highlighting the importance of preoperative planning for post-discharge support and proactive rehabilitation interventions to help patients maintain independence at home after discharge.

Our findings suggest that MIS may help reduce the risk of LOI in elderly patients, as open surgery was identified as a significant risk factor. With an aging population, the number of elderly patients undergoing pancreatic surgery continues to rise, and MIS techniques such as laparoscopic and robotic approaches have been increasingly adopted. Although MIS is associated with longer operative times and specific complications such as subcutaneous emphysema, it has been reported to reduce intraoperative blood loss, shorten hospital stays, and facilitate earlier postoperative recovery. These advantages make MIS particularly meaningful for frail patients [20, 21]. Our results are consistent with previous reports and support the broader use of MIS in appropriate elderly candidates.

Sarcopenia was also identified as an independent risk factor for LOI in this study, emphasizing the need for preoperative physical assessment in elderly patients undergoing PD. While various methods exist for assessing muscle mass, such as psoas muscle area measurement on CT, these are often technically demanding and subject to interobserver variability [22, 23]. We utilized the InBody S10 device, which provides a simple, non-invasive, and reproducible evaluation of sarcopenia. This tool has been widely applied in clinical fields such as geriatric medicine, surgery, and orthopedics [24–26], allowing for early identification of sarcopenia and timely nutritional interventions that may reduce the risk of LOI. Although frailty has been studied as a risk factor for postoperative LOI after gastrectomy, hepatectomy, and so on, no reports have specifically addressed PD. Frailty is an important concept, so we analyzed sarcopenia, a physical component of frailty and an objective indicator, as a risk factor for LOI. This study is the first to examine LOI as an outcome in PD, which is critically essential in clinical practice. It demonstrates that preoperative sarcopenia is one of the risk factors, thereby complementing existing frailty research.

To facilitate risk stratification, we developed a simple LOI scoring system based on the presence of three identified risk factors: age ≥ 80 years, sarcopenia, and open surgery. The score was intentionally simplified so that it can be applied by multidisciplinary teams, and it is composed entirely of preoperative factors, allowing for risk assessment at an early stage. A score of 0 or 1 was associated with a low incidence of LOI, while a score of 2 or more indicated a significantly higher risk. For patients with higher scores, thorough preoperative and postoperative discussions about the possibility of LOI are warranted, and the use of minimally invasive surgical approaches should be considered whenever feasible. Although a nomogram could have been used to provide weighted risk estimates, we opted for a simple additive scoring model to improve accessibility for both medical and non-medical personnel. Moreover, by anticipating the potential risk of LOI, healthcare teams can proactively implement individual perioperative support, including enhanced rehabilitation, nutritional interventions, and coordinated social or home-based care planning. This facilitates smoother transitions after surgery and may help patients maintain independence throughout recovery. Compared with previous LOI prediction tools, our model showed higher discrimination while remaining simple. Sakurai et al. [27] developed a model for elderly gastric cancer patients (≥ 65 years) using age ≥ 75 years, frailty, and Clavien–Dindo grade ≥ 3 complications (AUC = 0.765). Tanaka et al. [11] assessed LOI risk after hepatectomy using age ≥ 76 years, frailty, and open surgery (AUC = 0.777 in the study cohort; 0.783 in the validation cohort). In contrast, our PD-specific model, composed of age ≥ 80 years, objectively measured sarcopenia, and surgical approach, achieved an internally validated AUC of 0.873, indicating both high predictive accuracy and clinical applicability.

The proportion of elderly individuals in the global population is expected to continue rising until at least 2060, contributing to a marked increase in elderly patients undergoing major abdominal surgeries such as PD [28, 29]. As a result, preoperative identification of patients at high risk for LOI has become increasingly important. The present study highlights the need to consider MIS whenever feasible, particularly in sarcopenic elderly patients. The finding that open surgery is a significant risk factor for LOI may contribute to improving surgical decision-making and further promoting the development and appropriate application of minimally invasive approaches in pancreatic surgery. In addition, Enhanced Recovery After Surgery (ERAS) protocol [30, 31], which emphasizes early mobilization, optimal pain control, and nutritional support, may further enhance recovery and prevent LOI.

This study has several limitations. Firstly, it was a retrospective, single-center analysis with a relatively small proportion of elderly patients. In addition, as this study involved a two-group comparison, we believe that a formal multiple comparison adjustment was not essential, and therefore it was not performed. Secondly, the long-term functional outcome beyond 90 days was not evaluated. Socioeconomic status and caregiver support may also have been confounding factors. In Japan, universal health insurance covers all medical and nursing care services regardless of these factors, which may limit the generalizability of our findings to other healthcare systems. Although LOI was consistently defined based on standardized criteria, its external applicability should be interpreted with caution. Furthermore, preoperative assessment of sarcopenia may be challenging. The InBody method is not always feasible, as some institutions do not have the device or cannot perform the assessment due to scheduling constraints, and it cannot be used in patients with a pacemaker. We described 11 patients who were excluded from the analysis due to the lack of preoperative sarcopenia assessment (Table S3). Although sarcopenia can also be assessed using alternative methods such as CT-based muscle mass measurement, we routinely measured sarcopenia using the InBody method during nutritional counseling by dietitians. Because such counseling itself could be a potential confounding factor, patients who did not undergo InBody assessment were excluded from the analysis. Finally, the MIS group included fewer malignant cases and tended to comprise younger patients, suggesting a potential selection bias. However, malignant disease was not an independent risk factor for LOI, and there were no significant differences in vascular or multivisceral resections, age ≥ 80 years, or sarcopenia between MIS and open groups. Therefore, it reflects real-world clinical practice, and its impact on this study is considered limited. Future multicenter prospective studies with larger sample size are warranted to validate and refine these findings.

In conclusion, we firstly reported three independent risk factors of LOI after PD: age ≥ 80 years, sarcopenia, and open surgery. The proposed risk assessment tool is simple and practical, with strong clinical utility, particularly in preoperative decision-making and surgical planning. For sarcopenic patients aged ≥ 80 years, MIS performed by experienced surgeons may help reduce the risk of LOI and support postoperative independence in elderly patients undergoing PD.

## Supplementary Information

Below is the link to the electronic supplementary material.

Supplementary file1 (DOCX 19 KB)

Supplementary file2 (TIFF 96 KB)

Supplementary file3 (TIFF 316433 KB)

Supplementary file4 (TIFF 316433 KB)
