# Supplementary material for: Minimally invasive surgery reduces the risk of loss of independence after pancreatoduodenectomy in elderly patients
Source: Surg Endosc. 2025 Dec 29;40(3):2332–40. doi: 10.1007/s00464-025-12518-2 (PMC12971829; doi:10.1007/s00464-025-12518-2)
Supplement: Supplementary file 1 — Supplementary file1 (DOCX 19 KB) [file 464_2025_12518_MOESM1_ESM.docx]

**Supplementary Table 1: Loss of independence patient list**

| Patient No | Age | Sex | Sarcopenia | Diagnosis | Malignancy | Neoadjuvant Chemotherapy | LOI Score | LOI |
| --- | --- | --- | --- | --- | --- | --- | --- | --- |
| 1 | 70 | M | No | IDCs | Yes | No | 1 | Home Based Healthcare |
| 2 | 70 | M | No | BDC | Yes | No | 1 | Home Based Healthcare |
| 3 | 80 | F | No | IDCs | Yes | No | 2 | Rehabilitation Transfer |
| 4 | 76 | F | Yes | BDC | Yes | No | 2 | Home Based Healthcare |
| 5 | 74 | M | Yes | NET | No | No | 2 | Home Based Healthcare |
| 6 | 70 | M | Yes | IDCs | Yes | Yes | 2 | Home Based Healthcare |
| 7 | 70 | M | Yes | IDCs | Yes | Yes | 2 | Home Based Healthcare |
| 8 | 73 | M | Yes | Others | Yes | No | 2 | Home Based Healthcare |
| 9 | 88 | F | Yes | SPN | No | No | 3 | Rehabilitation Transfer |
| 10 | 87 | F | Yes | IPMN | No | No | 3 | Readmission for poor functionality |
| 11 | 84 | F | Yes | IDCs | Yes | No | 3 | Home Based Healthcare |
| 12 | 83 | F | Yes | IDCs | Yes | No | 3 | Readmission for poor functionality |
| 13 | 85 | M | Yes | BDC | Yes | No | 3 | Home Based Healthcare |
| 14 | 84 | M | Yes | BDC | Yes | No | 3 | Home Based Healthcare |
| 15 | 83 | M | Yes | IDCs | Yes | No | 3 | Home Based Healthcare |
| 16 | 79 | M | Yes | IPMN | No | No | 2 | Home Based Healthcare |
| 17 | 79 | M | No | BDC | Yes | No | 1 | Home Based Healthcare |
| 18 | 81 | M | Yes | IDCs | Yes | No | 3 | Readmission for poor functionality |
| 19 | 80 | F | No | Vater C | Yes | No | 2 | Home Based Healthcare |
| 20 | 81 | M | Yes | BDC | Yes | No | 3 | Home Based Healthcare |
| 21 | 73 | M | Yes | Duo | Yes | Yes | 2 | Home Based Healthcare |
| 22 | 75 | M | Yes | IDCs | Yes | No | 2 | Home Based Healthcare |

IDCs: Invasive ductal carcinoma

BDC: Bile duct cancer

NET: Neuroendocrine tumor

IPMN: Intraductal Papillary Mucinous Neoplasm

Vater C: Ampullary cancer

**Supplementary Table 2: Clinical characteristics and surgical outcomes in the elderly patients comparing Open Surgery (n=106) vs MIS (Laparoscopic n=12, Robotic n=12)**

| **Variables** | Open Surgery (n=106) | **MIS (n=24)** | P |
| --- | --- | --- | --- |
| *Patient characteristics* |  |  |  |
| Age, years; median (range) | 74 (65-88) | 71.5 (65-82) | 0.046 |
| Age ≥ 80 years | 17 (16.0) | 2 (8.3) | 0.524 |
| Sex (male/female) | 63/43 | 15/9 | > 0.999 |
| BMI, kg/m^2^; median (range) | 22.0 (15.8-29.8) | 23.3 (15.3-28.8) | 0.547 |
| ASA score ≥3 (%) | 18 (17.1) | 5 (20.8) | 0.768 |
| Sarcopenia (%) | 35 (33.0) | 7 (29.2) | 0.812 |
| Primary disease |  |  |  |
| Malignancy (%) | 63 (59.4) | 8 (33.3) | 0.024 |
| Pancreatic cancer | 42 (38.9) | 4 (16.7) |  |
| Cholangiocarcinoma | 11 (10.4) | 1 (4.2) |  |
| Pancreatic neuroendocrine tumor | 8 (7.4) | 5 (20.8) |  |
| Intraductal papillary mucinous neoplasms | 29 (26.9) | 9 (37.5) |  |
| Papillary carcinoma | 8 (7.4) | 3 (12.5) |  |
| Others | 8 (7.4) | 2 (8.3) |  |
|  |  |  |  |
| *Preoperative factors* |  |  |  |
| Preoperative Blood test |  |  |  |
| WBC (/㎕) | 5500 (2500-9900) | 5150 (3200-7100) | 0.070 |
| Lymphocyte (/㎕) | 1372 (569-2867) | 1327 (414-2819) | 0.446 |
| Albumin (g/dL) | 3.9 (2.2-4.8) | 4.1 (3.3-4.6) | 0.023 |
| CRP (mg/dl) | 0.09(0.02-3.35) | 0.04 (0.02-3.35) | 0.006 |
| Prognostic Nutritional Index (PNI) | 46.5 (32.3-56.8) | 47.1 (40.4-54.1) | 0.166 |
| Neoadjuvant Chemotherapy (%) | 15 (14.2) | 2 (8.3) | 0.737 |
|  |  |  |  |
| *Surgery related factors* |  |  |  |
| Portal vein resection (%) | 12 (11.3) | 0 (0) | 0.122 |
| Colon resection (%) | 4 (3.8) | 0 (0) | > 0.999 |
| Operation time, min; median (range) | 479 (289-823) | 499 (383-876) | < 0.001 |
| Blood loss, ml; median (range) | 530 (115-5971) | 238 (40-1276) | < 0.001 |
| Blood transfusion (%) | 10 (9.5) | 0 (0) | 0.206 |
|  |  |  |  |
| *Postoperative complications* |  |  |  |
| Clavien-Dindo classification ≥ grade IIIa (%) | 35 (33.0) | 7 (29.2) | 0.812 |
| POPF (%) | 32 (30.5) | 7 (29.2) | > 0.999 |
| DGE (%) | 8 (7.5) | 2 (8.3) | > 0.999 |
| Post pancreatectomy hemorrhage (%) | 8 (7.5) | 3 (12.5) | 0.425 |
| Hospital stays, days; median (range) | 24 (14-109) | 26 (13-59) | 0.947 |
| Loss of Independence (%) | 22 (20.8) | 0 (0.0) | 0.013 |

MIS: minimally invasive surgery

WBC: White Blood Cell counts

CRP: C-reactive protein

BMI: body mass index

ASA: American Society of Anesthesiologist's physical status

PNI: prognostic nutritional index

POPF: postoperative pancreatic fistula

DGE: delayed gastric emptying

**Supplementary Table 3: Excluded cases list (Not evaluated sarcopenia)**

| Patient No | Age | Sex | Diagnosis | Malignancy | Open PD | LOI |
| --- | --- | --- | --- | --- | --- | --- |
| 1 | 81 | F | IDCs | Yes | Yes | No |
| 2 | 24 | F | Others | No | Yes | No |
| 3 | 75 | M | Vater C | Yes | Yes | No |
| 4 | 68 | F | Others | No | Yes | No |
| 5 | 68 | F | Others | No | Yes | No |
| 6 | 42 | M | IDCs | Yes | Yes | No |
| 7 | 79 | F | IDCs | Yes | Yes | No |
| 8 | 67 | F | Others | Yes | Yes | No |
| 9 | 78 | F | IPMN | No | Yes | No |
| 10 | 81 | F | Others | Yes | No | No |
| 11 | 61 | F | Vater C | Yes | No | No |

IDCs: Invasive ductal carcinoma

IPMN: Intraductal Papillary Mucinous Neoplasm

Vater C: Ampullary cancer

LOI: Loss of Independence
