# Supplementary figures and images for: Minimally invasive surgery reduces the risk of loss of independence after pancreatoduodenectomy in elderly patients
Source: Surg Endosc. 2025 Dec 29;40(3):2332–40. doi: 10.1007/s00464-025-12518-2 (PMC12971829; doi:10.1007/s00464-025-12518-2)

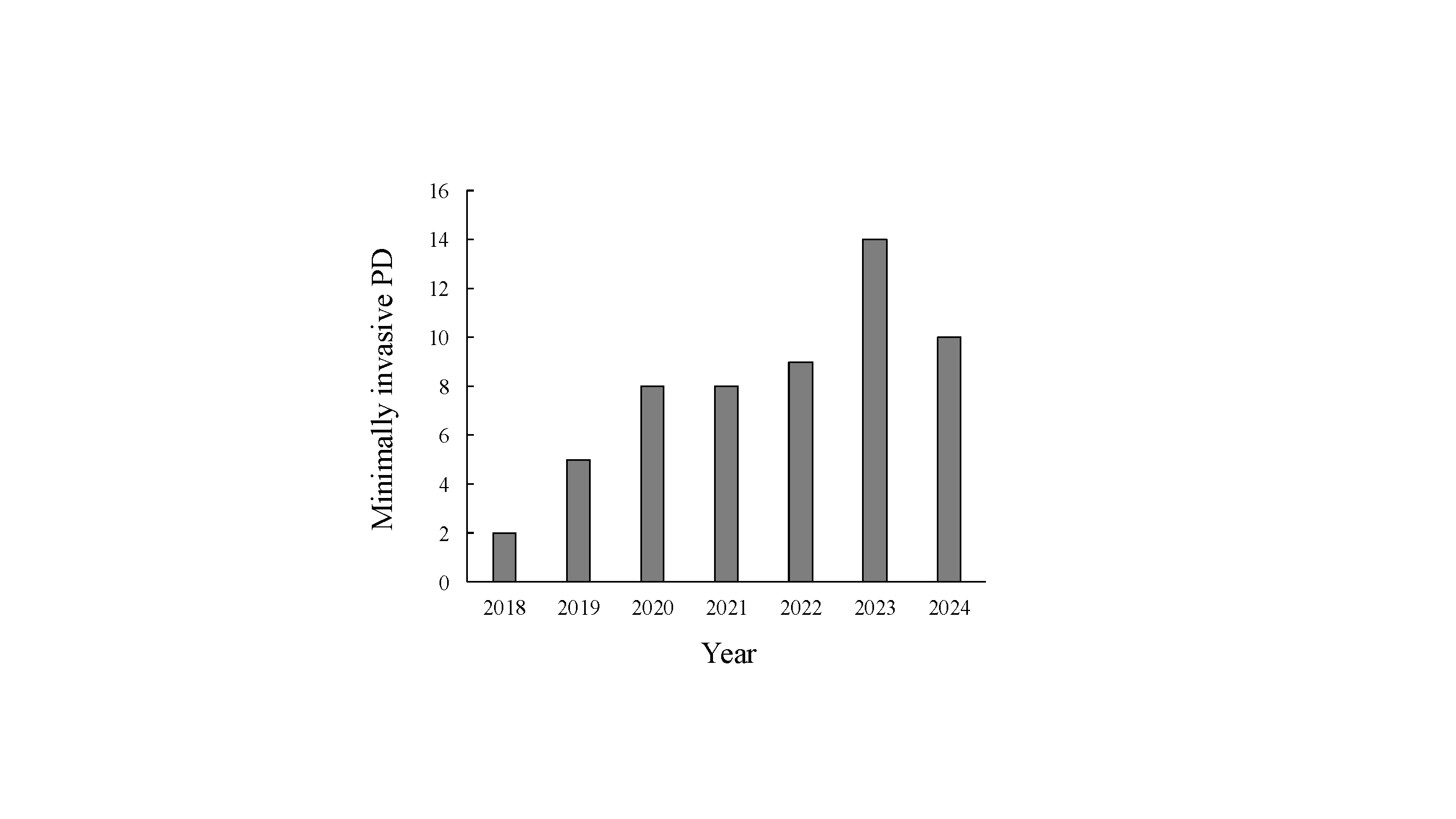

Supplement: Supplementary file 2 — Supplementary file2 (TIFF 96 KB) [file 464_2025_12518_MOESM2_ESM.tiff]
